# Resistance pattern and maternal knowledge, attitude and practices of suspected Diarrheagenic *Escherichia coli* among children under 5 years of age in Addis Ababa, Ethiopia: cross sectional study

**DOI:** 10.1186/s13756-018-0402-5

**Published:** 2018-09-12

**Authors:** Yeshwondm Mamuye GebreSilasie, Kassu Desta Tullu, Addisu Gize Yeshanew

**Affiliations:** 1 Department of Microbiology, St. Paul’s Hospital Millennium Medical College, P.O.Box 1271, Addis Ababa, Ethiopia; 2 Department of Microbiology, School of Medical Laboratory Sciences, College of Health Science, Addis Ababa University, Addis Ababa, Ethiopia

**Keywords:** Resistance patterns, Suspected diarrheagenic *E. coli*, KAPs

## Abstract

**Background:**

Diarrheal illness remains one of the leading causes of morbidity and mortality among children under 5 years of age worldwide, especially in developing countries. Diarrheagenic *Escherichia coli* (DEC) is the major cause of gastroenteritis in children in the developing world and is associated with high resistance levels to antibiotics. The aims of this study were to isolate and determine susceptibility patterns of DEC among children under 5 years of age with acute diarrhea and to assess maternal knowledge, attitude and practice towards childhood diarrhea.

**Methods:**

A cross sectional study was conducted from August–December 2015 at 3 selected health institutions. Stool samples were cultured and isolated *E. coli* species were run for antimicrobial susceptibility testing using disk diffusion method. In addition, children’s caretakers were interviewed using structured questionnaires including a Knowledge, Attitude and Practice (KAPs) survey. Bivariate and multivariate logistic regression analysis was used to quantify the effect of different risk factors on bacterial related diarrhea.

**Results:**

A total of 253 children, 115 males and 138 females with acute diarrhea were enrolled. *E. coli* was identified in a total of sixty-one children (24.1%), followed by *Shigella* (9.1%) and *Salmonella* (3.95%). Additionally, eighty-six children (34.0%) had parasites identified in stool samples. *E. coli* isolates showed 83.6% resistance to ampicillin and augmentin followed by, trimethoprim-sulfamethoxazole (62.3%). Multiple resistances were observed in 72.1% of isolates; however, more than 90% of the strains were sensitive to ciprofloxacin and ceftriaxone. Caretakers identified the following as causes of infection: contaminated food and water (83.4%), microorganisms (55.3%), inadequate breast milk (54.1%), teething (45.1%), house flies (43.1%) and evil eye (15.8%). No hand washing before meals and low levels of knowledge had a significant association with *E. coli* infection (*p* < 0.05).

**Conclusion:**

In children with suspected diarrheagenic *E. coli,* we observed a high frequency of multidrug resistant *E. coli*. Furthermore, study subjects with low awareness about source, cause and symptoms of the disease were more likely to acquire suspected diarrheagenic *E. coli* infections. Thus, there is a need for more education in addition to continuous surveillance of the prevalence and antibiotic susceptibility pattern of diarrheal bacterial isolates in hospitals and in the community.

## Background

Diarrhea is the third most common cause of deaths among children in sub-Saharan Africa and one of the main causes of hospital admissions in rural areas [1]. It is still considered one of the foremost causes of death in children, accounting for approximately 2 million deaths each year worldwide [2, 3]. In developing countries like Ethiopia, diarrheal diseases are major causes of infant and child mortality and morbidity [4].

Syndromes of diarrhea can be caused by bacterial, viral and parasitic infections of either single or multiple etiologic agents [5]. Suspected Diarrheagenic *Escherichia coli* (DEC) are considered to be the most common of the many recognized enteropathogenic organisms, particularly in developing countries and in diarrhea-associated deaths in children under five [6].

Moreover, the emergence of antimicrobial-drug resistance, including resistance to the new and potent antimicrobial agents, is a major public health concern especially in resource-limited countries, like Ethiopia, where bacterial infections are still among the major causes of death, especially for children, it is of particular concern [7, 8].

Anti-diarrheal, anti-amoebic and anti-bacterial medications have little role in the management of diarrhea, on the basis of WHO guidelines [9]. However, timely management of the children with fluids resuscitation has substantially declined the mortality and morbidity from acute infectious diarrhea [3]. In Ethiopia, evidence is lacking regarding maternal care-taking and environmental risk factors that contribute to acute diarrhea and the case management of diarrhea. Thus, in view of increasing diarrheal disease and emergence of antimicrobial resistance internationally, there is a need identify misconceptions or misunderstandings about transmission of diarrheal illness in the community in addition to determining resistance patterns of isolated bacterial pathogens involved in diarrheal illness. A Knowledge, Attitude and Practices (KAP) survey is a quantitative method that provides access to quantitative and qualitative information used in multiple studies to understand gaps in knowledge. Therefore, this study provides important input on the emergence of antimicrobial resistance and maternal KAPs to design appropriate control measures for Ethiopian children. This study also will improve awareness to clinicians and local communities in terms of potential targets for intervention strategies.

## Methods

### Study area and population

The study was conducted at outpatient pediatric departments of selected health institutions (Selam health center, Addis Ketema health center, and St. Paul’s Hospital Millennium Medical College) in Addis Ababa, Ethiopia from August–December 2015. Addis Ababa is the capital and largest city of Ethiopia. Addis Ababa had an estimated population of 3,384,569 according to the 2007 population census, with annual growth rate of 3.8%. These numbers were initially underestimated, as such; there are no accurate demographic data on Addis Ababa. However, more than 10% of the Ethiopian population can be attributed to children under five years of age.

### Sample size and sampling procedures

The sample size for the study was calculated using the formula (*n* = (zα/2)2 p (1-p)/ d2) for estimating a single population proportion at 95% confidence interval (CI) (Zα/2 = 1.96), 5% margin of error. Therefore, based on previous prevalence of *E. coli*, 20.8% study in other health facilities, the total sample size is 253 [10]. This sample size was also applied for caretakers to investigate their KAP. To get those study subjects, we utilized a randomly sampling techniques in which patients from the 3 selected heatlh institutes were our sampling frame (the population from which the samples were chosen). Specifically, we reviewed the daily data about patient flow from the selected health institutions to get the total of *n* = 253 subjects or to achieve our total sample size. Consequently, if an average daily patient flow to the pediatric department was = 30, then our sampling fraction become (253/30 = 8.4) that means almost 8. Then we selected random number from between 1 and 8, for example if that number is 3, the 1st selected study individual will be 3, then the 2nd will be (3 + 8 = 11), then the 3rd will be (11 + 8 = 19, then the 4th one will be (19 + 8 = 27)…., will continue the pattern of sequence by jumping 8 individuals until the selected participants will become total of 253 study subjects (3,11,19,27,35,43,51,59,67,75,83, etc., will be selected individual for the study until the sum of all selected individuals equaled 253 subjects).

### Data collection

#### Stool sample collection

We followed the same study protocol as previously published work for resistance patterns of *Shigella* and *Salmonella spp* among under 5 children with acute diarrhea [11]. Trained nurses used a pretested questionnaire to first interview the mother or primary caretaker. The mother or primary caretaker was then given a clean plastic stool container and oriented about sample collection. Once collected, protozoa parasites were identified through direct microscopy using a saline wet mount at each study sites by experienced laboratory technicians/technologists. Part of the stool was kept in Cary-Blair transport media, and transported in an icebox to the microbiology department of St. Paul’s Hospital Millennium Medical College (SPHMMC) for further microbiological investigations.

#### Culture identification and antimicrobial drug susceptibility test methods

Enteric pathogen underwent overnight incubation with Selenite-F Broth enrichment media for further multiplication. This was then subsequently sub-cultured onto MacConkey agar (MAC), Salmonella-Shigella agar (SSA) and Deoxycholate citrate agar (DCA), and then incubated aerobically at 37 °C for 24 h. After overnight incubation, colonies that exhibited characteristics of *Salmonella* and *Shigella* species were identified by conventional biochemical methods [12]. Because of many enteric bacteria can also grow in the enrichment broth, further detection techniques were performed to ensure specific isolation of *E. coli* colonies. Suspicious colonies were transferred to Triple Sugar Iron (TSI) agar, tryptone broth, arabinose broth, and urea broth and incubated for 20 h at 35 °C. Then H_2_S-positive, urease-positive, arabinose non-fermenting and indole-negative strains were rejected. Both *E. coli* and *Shigella* are anaerogenic (i.e. produce little or no gas) and non-motile, therefore to differentiate *E. coli* from *Shigella*, slow lactose fermenters were examined for lysine decarboxylase, mucate, and acetate reactions. *Shigella sonnei* was characterized based on a negative indole reaction and slow or non-fermentation of lactose [12, 13].

Shortly, colonies were first examined for lactose fermentation on MacConkey agar. The oxidase and indole tests were performed from a companion blood agar plate. *E. coli* strains were identified as lactose fermenting, betaglucuronidase and indole positive, and oxidase negative. Once suspected diarrheagenic *E. coli* and other enteric bacteria were isolated, drug susceptibility testing was performed using disc diffusion method. Susceptibility testing for all isolates was done and interpreted on the basis of CLSI guidelines [12]. Quality control was performed to check the quality of the medium, the potency of the antibiotic, and assay setup errors. Each new lot was quality controlled before use by testing the *E. coli* ATCC 25922 standard strains as we describe it previously [11–14].

#### Maternal knowledge, attitude and practices

A structured questionnaire was designed to collect information regarding socio-demographics and KAP of the study participants about diarrhea. The questionnaire was first developed in English and translated into Amharic (the local language), and then pre-tested in non-selected health institutions via pilot study for assessing content validity, appropriateness, and question comprehensibility. Then, the questionnaire was revised when necessary. A total of three nurses, one from each selected study area were selected to collect data. Training was given to the data collectors for two days on how to conduct the interview, content of the questionnaire, data quality, and ways to approach respondents. The first author checked the questionnaires for completeness every day. Incomplete questionnaires were returned to data collectors for correction by revisiting the caretakers. Five percent of the interviewed caretakers were randomly selected and re-interviewed by the first author. The KAP survey contains a total of 33 items. Thirteen items were knowledge related questions. Each correct response was assigned score 1 and wrong response was assigned 0 (zero). Thus for 13 items, the maximum attainable score was 13 and minimum was 0. Nine rating-scale items were for measuring the attitude of caretaker towards childhood diarrhea. The positive items were scored as 1 = strongly disagree, 2 = disagree, 3 = neutral, 4 = agree, and 5 = strongly agree. Scoring was reversed for negative items. Eleven items were for measuring the practice of the mother.

#### Operational definition

Suspected diarrheagenic *E. coli* (DEC) in this study was defined as a strain of *E. coli* spp. isolated from children under five years with acute diarrhea and considered as pathogenic.

Diarrhea was defined as at least three loose stools in 24 h including at least one of nausea, vomiting, abdominal cramps or fever symptoms.

The level of Knowledge, Attitude and Practices was classified according to the following: a poor knowledge corresponded to a score below 50%, and good knowledge referred to a score ≥ 50%. A score ≥ 50% was a “positive level” and a score of < 50% was classified as “negative level” of attitude of caretakers toward childhood diarrhea. A score < 50% was “poor” and a score ≥ 50% was considered as “good” level of practice.

#### Data analysis

Data were double entered, cross-checked using Epi-data version 3.3, and analyzed using SPSS version 20. Enteric bacteria prevalence was determined by dividing the number of individuals infected with enteric bacteria by the total number of individuals examined for bacterial infection. Frequency distribution tables were used to quantify enteric bacterial and parasitic infection, in addition to knowledge of respondents related to sign symptoms, causes, transmission, prevention and control measures of diarrhea. Logistic regression was used to examine the association between independent predictors and dependent enteric bacterial infection. Bivariate and multivariate logistic regression analysis was used to quantify the effect of different risk factors including KAP on bacterial related diarrhea. Values were considered statistically significant at *p* < 0.05.

#### Ethical considerations

The study obtained ethical clearance from the Department Research and Ethical Review Committee (DRERC) of Addis Ababa University School of Allied Health Sciences, Department of Medical Laboratory sciences and each of the selected health institutes. Written informed consent was obtained from voluntary participants and parents or primary caretakers for children during data collection. Individuals who were found positive for bacteria and parasite were treated as per the national guidelines.

## Results

### Socio-demographic characteristics of children and prevalence of diarrhea

As mentioned, this is a part of work from the previous published study [11]. A total of 253 primary caretakers were interviewed and provided stool samples from children for enteric bacterial and parasite identification. The mean age of the patients was 2.61 years with standard deviation (SD) of 1.26 years. In regards to caretakers, most of respondents (87%) were females (*n* = 220). For all respondents, 66.0% were between 26 and 40 years, ranging from 16 to 52 years with a median of 29 years; 86.2% were married (*n* = 218), and 64.9% had completed a primary education (*n* = 164). The majority of caretakers (53.4%) were house wives (*n* = 135); and the income for 47% of caretaker’s (*n* = 119) was less than 500 ETB (18 USD) per month. 5.5% of the caretakers had completed secondary or a higher level of education (Table 1).

**Table 1:** Distributions of socio-demographic characteristics of children and caretakers at the selected public health institutions in Addis Ababa, Ethiopia, 2015

Study Subjects	Variable	Category	Frequency, *n* = 253	Percent
	Sex	Male	115	45.5
		Female	138	54.5
Children		< 1 yrs	32	12.6
	Age	1-2 yrs	72	28.5
		2-3 yrs	64	25.3
		> 3 yrs	84	33.6
	Sex	Male	33	13.0
		Female	220	87.0
	Age	≤25 yrs	78	30.0
		26-40 yrs	167	66.0
		≥41 yrs	8	3.2
Caretakers	Marital Status	Single	15	6.0
		Married	218	86.2
		Divorced	12	4.7
		Other	8	3.1
	Educational Status	Illiterate	42	16.6
		Primary	164	64.9
		Secondary	33	13.0
		Higher education	14	5.5
	Occupation	Government	25	9.9
		Merchant	63	24.9
		Housewife	135	53.4
		Others	30	11.8
	Monthly Income	< 500 ETB	119	47.0
		501–1000 ETB	41	16.2
		1001–1500 ETB	12	4.8
		> 1500 ETB	5	2.0
		Others	76	30.0

*ETB* Ethiopian Birr

Enteric bacteria were isolated from 94 (37.2%) of the children tested. The predominant isolated organisms were suspected diarrheagenic *E. coli* spp. (*n* = 64 or 24.1%), followed by *Salmonella* species (*n* = 23 or 9.1%) and *Shigella* species (*n* = 10 or 3.95%). 34% of samples (*n* = 86) were positive for parasites; the most frequently identified protozoan parasites were *E. histolytica/dispar* (*n* = 45 or 17.8%), followed by *G. lambia* (*n* = 26 or 10.3%), *H. nana (n =* 9 or 3.6%), *A. lumbricoides* (*n* = 5 or 2.0%) and *S. stercolaris* (*n* *=* 1 or 0.4%).

### Antimicrobial sensitivity results of isolates

The antimicrobial susceptibility testing was done for all suspected diarrheagenic *E. coli* isolates using disk diffusion method. Zones of inhibition were measured by using caliper meter and interpreted as sensitive (S), intermediate (I), and resistance(R) respectively on the basis of CLSI guide lines [14].

Suspected diarrheagenic *E. coli* (DEC) were isolated from 24.1% of stool samples. Among patients who had suspected diarrheagenic *E. coli* infections, the resistance rates were high for ampicillin (83.6%), augmentin (83.6%), trimethoprim-sulfamethoxazole (62.3%), and medium for chloramphenicol (21.3%), nalidixic acid (19.7%), and gentamicin (11.5%). Low levels of resistance were observed against ciprofloxacin (4.9%) and ceftriaxone (3.3%) (Table 2).

**Table 2:** Antimicrobial susceptibility patterns of *E. coli* isolates among children under five years of age at the selected public health institutions in Addis Ababa, Ethiopia, 2015

Enteropathogen (n)	Interpretation	Antibiotics							
		Amp	Aug	SXT	C	CIP	Gen	Na	CRO
Suspected Diarrheagenic *E. coli* (61)	S (%)	11.5	9.8	36.1	72.1	93.4	63.9	73.8	96.7
	I (%)	4.9	6.6	1.6	6.6	1.6	24.6	6.6	0
	R (%)	83.6	83.6	62.3	21.3	4.9	11.5	19.7	3.3

^*^S sensitive, *I* intermediate, and *R* resistant, *Amp* Ampicillin, *Aug* Augmentin, *SXT* trimethoprim-sulfamethoxazole, *C* Chloramphenicol, *NA* Nalidixic Acid, *CIP* Ciprofloxacin, *Gen* Gentamicin, *CRO* Ceftriaxone, *n* number

Antimicrobial resistance to one or more antibiotics was very high among the suspected diarrheagenic *E. coli* species isolated in the study (83.6%). Multiple resistances (resistance for two or more commonly used antibiotics) were observed in 85.2% of the DEC species isolated. One of the isolated strains of suspected diarrheagenic *E. coli* was resistant for seven antibiotics. Furthermore, susceptibility to all eight antibiotics tested was observed for 3 (4.9%) of the isolates.

### Knowledge, attitude and practice (KAP) towards diarrhea

Out of 253 respondents, 83.4% (*n* = 211) had heard of diarrhea, and 70.8% (*n* = 179) of them mentioned diarrhea as one of the major health problems of the community. 97.9% of caretakers (*n* = 247) used pipe water as the primary source of water in home. However, two patients used stagnant water sources and both were positive for enteric pathogens. On bivariate analysis, low income, < 500 ETB (OR = 3.77, 95% CI = 1.454–9.77, *P*-value = 0.006), 500–1000 ETB (OR = 6.47, 95% CI = 1.922–21.775, *P*-value = 0.003) and absence of hand washing before and after meal (OR = 0.12, 95% CI = 0.02–0.062, *P*-value = 0.03) were important independent predictors of suspected diarrheagenic *E. coli* infections. However, multivariate logistic analysis revealed that only poor hand washing practice had a statistically significant association with detection of suspected diarrheagenic *E. coli* infection. Children from households that wash their hands before and after meals are 81% less likely to have DEC infection compared to children from households who don’t wash their hands (AOR = 0.19, 95% CI = 0.144–0.775, *P*-value = 0.004) (Table 3).

**Table 3:** Associations of risk factor with culture positivity of *E. coli* at the selected public health institutions in Addis Ababa, Ethiopia, 2015

Variables	Suspected Diarrheagenic *E. coli*					*P*-Value
	Negative, N (%)	Positive, N (%)	COR (CI)	*P*-Value	AOR (CI)	
Marital Status						
Never Married	12(75.0)	4(25.0)	1	0.93		
Ever Married	180(75.9)	57(24.1)	0.95(0.29–3.1)			
Educational status						
Illiterate	29(69.0)	13(31.0)	1.28(0.17–9.9)	0.33		
Primary school	126(76.8)	38(23.2)	0.73(0.09–5.8)	0.51		
Secondary school	26(78.8)	7(21.2)	1.06(0.15–7.8)	0.40		
Higher education	11(78.6)	3(21.4)	1			
Occupation						
Government	19(76.0)	6(24.0)	1			
Merchant	49(77.8)	14(22.2)	0.19((0.2–1.9)	0.30		
Housewife	101(74.8)	34(25.2)	0.38(0.4–3.5)	0.41		
Others	23(76.7)	7(23.3)	3.12(0.3–30.7)	0.33		
Monthly Income						
< 500	98(72.1)	38(27.9)	3.8(1.5–9.8)	0.006*	0.12(−0.05-001)	0.060
501–1000	37(69.8)	16(30.2)	6.5(1.9–21.8)	0.003*	0.22(0.64-1.3.)	0.056
1001–1500	27(84.4)	5(15.6)	1.3(0.12–12.2)	0.61	1.13(0.22–5.74)	0.880
> 1501	30(93.8)	2(6.2)	1			
Family previous diarrhea						
Yes	22(71.0)	9(29.0)	1.47(0.53–1.4)	0.64		
No	170(76.6)	52(23.4)	1			
Attending day care						
Yes	73(79.3)	19(20.7)	1			
No	119(73.9)	42(26.1)	1.3(0.7–2.4)	0.35		
Feeding practice						
Exclusive breast milk	35(89.7)	4(10.3)	1			
Breast milk & solid food	58(72.5)	22(27.5)	3.5(1.07–11.0)	0.07		
Solid food only	95(73.6)	34(26.4)	3.5(1.1–11.2)	0.08		
Formula Milk	4(80.0)	1(20)	2.6(0.23–34.4)	0.28		
Proper usage of latrine						
Yes	187(76.0)	59(24.0)	1			
No	5(71.4	2(28.6)	0.7(0.05–11.6)	0.83		
Raw meat usage						
Yes	111(78.2)	2(8.7)	0.26(0.06–1.2)	0.08		
No	81(73.0)	59(25.7)	1			
Hand washing before & after meal						
Yes	190(77.2)	56(22.8)	1			0.004*
No	2(28.6)	5(71.4)	0.12(0.02–0.6)	0.03*		0.2(0.2-8)
Sources of water						
Pipe	188(76.1)	59(23.9)	1			
Other	4(66.7)	2(33.3)	1.6(0.29–8.9)	0.60		
Knowledge						
Good	168(79.2)	44(20.6)	1			
Poor	24(58.5)	17(41.5)	2.65(1.3–5.4)	0.008*	2.70(1.33-5.5)	
Attitude						
Positive	173 (77.6)	50(22.4)	1			
Negative	19 (63.3)	11(36.7)	1.8(0.88–4.11)	0.163		
Practice						
Good	181(76.1)	57(23.9)	1			
Poor	11(73.3)	4(26.7)	1.4(0.4–4.7)	0.582		

^*^Statistically significant*, CI* 95% confidence interval*, COR* crude odds ratio*, AOR* adjusted odds ratio

Adjusted OR (adjusted odds ratio from multivariable logistic regression model) = when the effect of one factor on E. coli prevalence is evaluated the analysis was adjusted for other remaining factors listed in the table

From a total of 253 respondents, 158 (62.5%) of them stated diarrhea as a common childhood illness in their area. 225 (88.9%) respondents associated the sources of children’s diarrheal infection with poor personal and environmental hygiene (Table 4).

**Table 4:** Distributions of respondents according to knowledge and attitude towards the symptom and cause of childhood diarrhea at the selected public health institutions in Addis Ababa, Ethiopia, 2015

Cause, Symptoms and prevention of diarrhea	Variable	Frequency (%)
Cause of childhood diarrhea mentioned	Poor personal hygiene	225 (88.9)
	Contaminated food/water	211 (83.4)
	Microorganisms	140 (55.3)
	Inadequate breast milk	137 (54.1)
	Teething	116 (45.8)
	Evil eye	40 (15.8)
	House flies	109 (48.1)
	Do not know	6 (2.4)
Sign/symptoms of childhood diarrhea	3–4 times loose stool per day	234 (92.5)
	Abdominal pain	198 (78.3)
	Fever	165 (65.2)
	Vomiting	140 (55.3)
	Tenesmus	134 (53.1)
	Do not known	9 (3.6)
Cause to worsens childhood diarrhea	Contact with infected people	228 (90.1)
	High fluid intake	122 (48.2)
	Formula milk	120 (47.4)
Preventive methods	Oral rehydration solutions	240 (94.9)
	Personal hygiene	237 (93.7)
	Breast milk	224 (88.1)
	Clean water intake	162 (64.0)
	Anti-diarrheal treatment	123 (48.6)
	Antibiotics	53 (20.9)
	Traditional medicine	22 (8.7)
	Do not know	12 (4.7)

Note: Percentages do not add up to 100 because of multiple responses

The majority of respondents, 92.5% (*n* = 234) and 78.3% (*n* = 198) mentioned frequent diarrhea and abdominal pain as important and noticeable symptoms of childhood diarrhea, respectively. However, a few (3.6%) participants were not aware of the signs and symptoms.

Regarding their practice, the majority of the caretakers went to health centers, and no one sought out traditional healers. 91.7% (*n* = 232) participants went to health centers, 7.5% (*n* = 19) went to hospitals, and 0.8% (*n* = 2) went to private clinics for medical treatment. Any fluid intake, defined as taking of any fluid drinks in addition to oral rehydration solutions, was noted as a control mechanism in 89.3% (*n* = 226) of respondents. Consequently, more than 90% of the caretakers used oral rehydration solution (ORS) as a treatment option (Table 5).

**Table 5:** Practices of respondents towards childhood diarrhea prevention and control at the selected public health institutions in Addis Ababa, Ethiopia, 2015

Variables	Frequency (%)
Exclusive breast feeding	234 (92.5)
Oral rehydration solutions	230 (90.9)
Any fluid intake in addition to ORS	226 (89.3)
Anti-diarrheal treatment	31 (12.3)
Antibiotics treatment	13 (5.1)
Traditional medicines	1 (0.4)
Do not use	2 (0.8)

Note: Percentages do not add up to 100 because of multiple responses

56.1% (*n* = 142) and 9.1% (*n* = 23) of caretakers used raw milk and raw meat, respectively. However, there was no statistically significant association detected between users and non-users with culture positivity. Of all respondents, 97.2% (*n* = 246) endorsed proper latrine usage. In general, children from caretakers with a low knowledge score were three times more likely to be exposed to *E. coli* infection than children from caretakers with a good knowledge score.

## Discussion

The overall isolation prevalence rate of suspected diarrheagenic *E. coli* in diarrheic stool samples was found to be 24.1%. This result was consistent with studies done in Ethio-Swedish Children’s Hospital, Addis Ababa Ethiopia, and Tanzania, where 20.8% and 22.9% rates of *E. coli* isolates have been reported respectively [10, 15]. However, our rates were lower compared to a 46.88% and 60.0% prevalence of suspected diarrheagenic *E. coli* from studies done in North West Italy and Nigeria, respectively [16, 17]. In contrast, our participants had higher rates of suspected diarrheagenic *E. coli* when compared to a study in the US of both inpatients (4.7%) and emergency room (10.0%) subjects [18]. Put together, the differences noted in the various populations might be due to age, socio-economic factors, and the nature of the public water supply.

83.6% of suspected diarrheagenic *E. coli* isolates were resistant to ampicillin (83.6%) and augmentin (83.6%). This in to contrast to other studies in other parts of Ethiopia, where ampicillin resistance was 93.70% [19]. This might be due to the difference in study design, place, age and period. The high prevalence of resistance to these drugs could be explained by the longtime use of this antibiotic to treat enteric bacterial infection, thereby ensuring selection pressure and maintenance of this resistance. Studies done in Peru, Iran, Nigeria, and Tanzania also show that diarrheagenic *E. coli* exhibited high levels of antimicrobial drug resistance [20–23].

In this study, multidrug resistance to two or more antibiotics (85.2%) was more commonly observed than resistance to a single drug (3.3%). Despite the high proportion of antimicrobial resistance observed among suspected diarrheagenic *E. coli* isolates, these organisms remain highly susceptible to ciprofloxacin and ceftriaxone, which are now the drugs of choice in many areas. This finding is in agreement with other studies done in Nigeria and Tanzania [18, 21–24].

A few of the caretakers had completed a higher level of education, and their children had a lower frequency of suspected diarrheagenic *E. coli* isolation. This is similar to a study showing a 54% risk reduction for severe disease among mothers with 7 or more years of education [25–27].

The features of acute diarrhea vary from place to place depending on local meteorology, geography, and socioeconomic variables [16]. However, common presenting features of suspected diarrheagenic *E. coli* include diarrhea that was watery or bloody, with or without mucus, fever, vomiting and abdominal pains. Similarly, in our cohort, the most useful signs and symptoms for the diagnosis of *E. coli* were the complaints of watery diarrhea, as reported in other studies [19].

Drinking of unsafe water and contaminated foods are often considered to increase exposure to enteropathogens and have been associated with increased rates of acute diarrhea [3]. This finding is contrary to a study in India that shows teething (64.3%), evil eye (46%), contact with another case (36.6%), worm infestation (22.6%), dirty water (15.3%), and dirty environment (6%) as causes [27].

Of all, 93.7% of the caretakers agreed that personal hygiene is an effective means to prevent childhood diarrhea and 88.5% mentioned breast milk. Moreover, during episodes of diarrheal illnesses, 92.5% (*n* = 234) of the mothers preferred breast milk. This attitude and practice is consistent with findings in Gondar, Ethiopia, that showed breast-feeding is protective factor [28]. This is also supported by other findings; breast-feeding especially if it is the only source of nutrition, has been shown to protect children against the development of diarrhea in Africa, as elsewhere in the developing world [3].

The majority of the caretakers went to health centers and no one consulted traditional healers. This is in agreement with studies done in other country [26]. 89% of mothers (*n* = 226) preferred fluids in contrast with other studies in Nepal where 15.7% preferred fluids [29]. Oral rehydration therapy was used as a treatment solutions for majority (90.9%) of caretakers, in contrast to other findings in India, where only 13(4.3%) preferred ORS [27]. This difference might be due to the educational status of the participant, as it is shown in the current study only 42 (16.6%) of the study subjects were illiterate as compared to 70% the previous study.

## Conclusion

We detected a high frequency of multi drug resistance suspected diarrheagenic *E. coli* in isolated bacteria in children under 5 with acute diarrheal illness. The majority of the participants’ caregivers had a good level of awareness when asked about the potential causes of diarrhea (e.g. which is mostly due to poor sanitation). Additionally, there was a statistically significant association detected between low levels of knowledge and suspected diarrheagenic *E. coli* culture positivity. This indicated that those respondents, who have low awareness about source, cause and symptoms of the disease were more likely to acquire suspected diarrheagenic *E. coli* infections. Thus, there is a need for further education in the community about how diarrhea illnesses are transmitted. Additionally, while our data suggest ciprofloxacin or ceftriaxone are good choices for empiric therapy of suspected diarrheagenic *E. coli*, antimicrobial resistance patterns can evolve. Hence, there is a still need for continuous surveillance of the prevalence and antibiotic susceptibility pattern of diarrheal bacterial isolates in hospitals and in the community.

Combined efforts should be also implemented to reduce childhood death rates by improving the knowledge, attitude, and practices level aimed at improving environmental (i.e. water sources) and personal hygiene (i.e. hand washing). Therefore, intensive health education is a public health priority that, together with more judicious use of antimicrobials, could preserve antimicrobial efficacy and substantially reduce diarrheal illness.

## Abbreviations

CI: Confidence Interval
CLSI: Clinical Laboratory Standard Institutes
DCA: Deoxycholate citrate agar
DEC: Diarrheagenic *Escherichia coli*
DRERC: Department Research and Ethical Review Committee
*E. coli*: *Escherichia coli*
ETB: Ethiopian Birr
KAPs: Knowledge, attitude, and practices
MAC: MacConkey agar
SPHMMC: St. Paul’s Hospital Millennium Medical College
SSA: *Salmonella Shigella* agar
WHO: World Health Organization.
